# Early Morbidity and Survival After Left Lateral Segment Versus Whole Liver Graft Transplantation in Children

**DOI:** 10.1111/petr.70501

**Published:** 2026-10-20

**Authors:** Helena Linge, Eva‐Doreen Pfister, Vanessa Muth, Sahar Arbabzadah, Jan Forner, Teresa Linge, Nicolas Richter, Markus Quante, Tung‐Yu Tsui, Ulrich Baumann, Moritz Schmelzle, Philipp Felgendreff, Cornelius J. Van Beekum

**Affiliations:** ^1^ Department of General, Visceral and Transplant Surgery Hannover Medical School Hannover Germany; ^2^ PRACTIS Clinician Scientist Program Hannover Medical School and the German Research Foundation Hannover Germany; ^3^ Hannover Medical School, Clinic for Pediatric Kidney, Liver, and Metabolic Diseases and Neuropediatrics Hannover Germany

**Keywords:** biliary complications, graft‐to‐recipient weight ratio, infants, left lateral segment, living donor liver transplantation, pediatric liver transplantation, split liver transplantation, surgical morbidity, whole liver graft

## Abstract

**Background:**

Left lateral segment (LLS) grafts expand graft availability for infants and small children requiring liver transplantation (LT). However, their cumulative early postoperative burden compared with whole liver grafts (WLGs) remains incompletely characterized.

**Methods:**

This retrospective single‐center cohort study included 95 consecutive primary pediatric LT recipients treated between 2019 and 2024, comprising 56 LLS and 39 WLG recipients. Cumulative postoperative morbidity within 90 days was quantified using the Comprehensive Complication Index (CCI). Additional outcomes included biliary and vascular complications, unplanned surgical reinterventions, length of stay, graft loss, retransplantation, and graft and patient survival.

**Results:**

LLS recipients were younger and smaller than WLG recipients. Cumulative 90‐day morbidity was greater after LLS transplantation (median CCI 53 vs. 45, *p* = 0.002). LLS recipients had higher rates of bile leakage (18% vs. 3%, *p* = 0.024), intra‐abdominal bleeding (15% vs. 0%, *p* = 0.019), and unplanned surgical reintervention (43% vs. 8%, *p* < 0.001). Although the overall incidence of biliary complications was similar between groups, anastomotic biliary strictures during follow‐up were more frequent after WLG transplantation (28% vs. 9%, *p* = 0.014). Hospital stay was longer after LLS transplantation (median 43 vs. 27 days, *p* < 0.001). No statistically significant differences in graft loss, retransplantation, or graft and patient survival were detected between groups.

**Conclusions:**

LLS transplantation was associated with greater cumulative morbidity within 90 days, characterized by higher rates of bile leakage, intra‐abdominal bleeding, unplanned surgical reintervention, and prolonged hospitalization. Despite this greater early postoperative burden, no statistically significant differences in graft loss, retransplantation, or graft and patient survival were detected during the available follow‐up. These findings support continued use of LLS grafts to expand transplant access. Given the associated early morbidity, it highlights the importance of an experienced surgical and medical team.

## Graphical abstract

Among 95 pediatric liver transplant recipients, LLS grafts carried greater early morbidity than whole liver grafts (CCI 53 vs. 45) but comparable graft and patient survival, supporting continued LLS use with an experienced surgical and medical team.

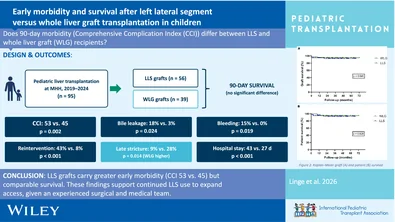

**Abbreviations**
ABOi: ABO‐incompatible
BA: biliary atresia
CCI: Comprehensive Complication Index
CDC: Clavien–Dindo classification
CI: confidence interval
CIT: cold ischemia time
ERCP: endoscopic retrograde cholangiopancreatography
GRWR: graft‐to‐recipient weight ratio
HAT: hepatic artery thrombosis
HJ: hepaticojejunostomy
HU: high urgency
ICU: intensive care unit
INR: international normalized ratio
IQR: interquartile range
LDLT: living donor liver transplantation
LLS: left lateral segment
LT: liver transplantation
MELD: model for end‐stage liver disease
OR: odds ratio
PELD: Pediatric End‐stage Liver Disease
PVT: portal vein thrombosis
RBC: red blood cell
reLT: retransplantation
SD: standard deviation
WIT: warm ischemia time
WLG: whole liver graft

## 1 Introduction

Liver transplantation (LT) is the definitive treatment for children with end‐stage liver disease, acute liver failure, and selected metabolic or malignant disorders. Although long‐term outcomes after pediatric LT are excellent, the limited availability of size‐matched donor organs remains a particular challenge for infants and small children. The scarcity of pediatric deceased donors may prolong waiting time and increase the risk of clinical deterioration or death before transplantation [1, 2].

Split liver transplantation and living donor liver transplantation have therefore become essential strategies for providing appropriately sized grafts to pediatric recipients [3, 4, 5]. Left lateral segment (LLS) grafts, comprising segments II and III, can be obtained either from deceased donors through liver splitting or from living donors. Their use has substantially expanded the availability of grafts for small children and has become an established component of contemporary pediatric transplant programs. In pediatric transplant practice, LLS grafts are used predominantly for infants and small children, whereas whole liver grafts (WLG) are generally transplanted into older and larger recipients; thus, both graft types serve complementary rather than competing indications. However, only a limited proportion of potentially suitable deceased donor livers are ultimately used for split liver transplantation [6, 7].

The advantages of LLS grafts must be considered alongside their technical complexity. Parenchymal transection, small‐caliber vascular and biliary structures, graft–recipient size mismatch, and the frequent need for staged abdominal or biliary reconstruction may increase the risk of postoperative complications and unplanned reinterventions. Previous studies comparing technical variants and WLG grafts have primarily focused on individual complications, graft loss, and patient survival [8, 9, 10, 11]. These outcomes may not fully reflect the cumulative postoperative burden experienced by children who undergo multiple interventions during the early postoperative period.

The CCI integrates all postoperative complications and their severity into a continuous measure and may therefore provide a more comprehensive assessment of early morbidity. We hypothesized that LLS transplantation is associated with a greater cumulative early postoperative burden than WLG transplantation, despite similar observed graft and patient outcomes. The main objective of this study was to compare cumulative postoperative morbidity within 90 days between pediatric recipients of LLS and WLG grafts.

## 2 Patients and Methods

### 2.1 Study Design and Patient Cohort

This retrospective single‐center cohort study included all LT procedures performed in recipients younger than 18 years at Hannover Medical School between January 2019 and December 2024. During the study period, 120 pediatric LT procedures were identified. The present analysis included primary LT using either an LLS graft or a WLG.

Monosegment grafts (*n* = 2), size‐reduced deceased donor grafts (*n* = 5), extended right lobe grafts (*n* = 2), retransplantations (*n* = 11), combined liver–kidney transplantations (*n* = 4), and combined liver–lung transplantation (*n* = 1) were excluded. After applying these criteria, 95 transplantations were included in the final analysis, comprising 56 LLS and 39 WLG recipients. All graft splitting procedures and transplantations were performed by the same experienced pediatric transplant team.

Clinical data were obtained from the institutional electronic medical records and included recipient and donor characteristics, underlying diagnosis, pretransplant clinical status, operative variables, postoperative complications, and follow‐up outcomes. Waiting time and allocation status were obtained from Eurotransplant records. Complete follow‐up data were available for all included recipients.

### 2.2 Ethics Statement

The study was conducted in accordance with the Declaration of Helsinki and was approved by the institutional ethics committee of Hannover Medical School (approval number 11651_BO_K_2024). Written informed consent for the use of anonymized clinical data for research purposes was obtained from the parents or legal guardians of all recipients. All data were anonymized before analysis.

### 2.3 Study Outcomes

The main outcome of interest was cumulative postoperative morbidity within 90 days after LT, quantified using the CCI.

Additional outcomes included biliary complications, hepatic artery thrombosis (HAT), portal vein thrombosis (PVT), major postoperative complications, unplanned surgical reinterventions, primary abdominal closure, length of intensive care unit (ICU) and hospital stay, graft loss, retransplantation, and patient mortality. HAT, PVT, bile leakage, and unplanned surgical reinterventions were assessed within 90 days after LT. Biliary strictures were evaluated over the entire available follow‐up period. Graft and patient survival were assessed from the date of LT until the respective event or last documented follow‐up.

### 2.4 Definition of Variables

Postoperative complications were graded according to the Clavien–Dindo classification. Major postoperative complications were defined as Clavien–Dindo grade IIIa or higher. The CCI incorporates all postoperative complications and their respective Clavien–Dindo grades into a continuous score ranging from 0, indicating no complications, to 100, indicating death. The CCI was calculated from all complications occurring within 90 days after LT using the validated online CCI calculator [12]. Bile leakage was defined according to the International Study Group of Liver Surgery as a bilirubin concentration in the drain fluid at least three times the serum bilirubin concentration on or after postoperative day 3 or the need for radiological or surgical intervention because of a biliary collection or bile peritonitis [13].

Biliary strictures were classified as anastomotic or non‐anastomotic and were defined as imaging‐confirmed biliary narrowing associated with biochemical evidence of cholestasis, graft dysfunction, or the need for endoscopic, radiological, or surgical treatment.

HAT and PVT were diagnosed by Doppler ultrasonography and confirmed by cross‐sectional or angiographic imaging when clinically indicated. An unplanned surgical reintervention was defined as any unscheduled reoperation required to diagnose or treat a postoperative complication. Planned second‐look procedures performed as part of a two‐step LT strategy were not classified as unplanned surgical reinterventions.

Two‐step LT was defined as a planned second‐look procedure for definitive biliary reconstruction and/or abdominal closure following graft implantation. Temporary abdominal closure was used when primary fascial closure was considered unsafe because of graft–recipient size mismatch, abdominal tension, or hemodynamic compromise.

The graft‐to‐recipient weight ratio (GRWR) was calculated by dividing graft weight in grams by recipient body weight in grams and expressing the result as a percentage.

### 2.5 Surgical Technique

All LT procedures were performed according to standardized institutional operative and perioperative protocols. Recipient hepatectomy and graft implantation were adapted to graft type, recipient anatomy, and previous abdominal surgery.

For LLS transplantation, recipient hepatectomy was performed using a cava‐preserving technique. Venous outflow was reconstructed using the recipient hepatic vein confluence, followed by end‐to‐end portal vein and hepatic artery anastomoses. Interposition or patch grafts were used when required by vessel size or anatomical considerations. Biliary reconstruction was performed using a Roux‐en‐Y hepaticojejunostomy. Depending on graft–recipient size mismatch, abdominal tension, and the recipient's clinical condition, biliary reconstruction was performed either during the initial transplantation or as part of a planned second‐look procedure. When primary abdominal closure was considered unsafe, temporary abdominal closure was followed by planned definitive fascial closure.

For WLG transplantation, recipient hepatectomy was generally followed by caval replacement with end‐to‐end anastomoses of the suprahepatic and infrahepatic inferior vena cava. Portal and arterial reconstruction was performed according to recipient and donor anatomy. In small, deceased donor grafts, arterial reconstruction could include an end‐to‐side anastomosis of the donor aorta to the supraceliac recipient aorta. Biliary reconstruction was performed either by duct‐to‐duct anastomosis or Roux‐en‐Y hepaticojejunostomy, as clinically appropriate.

After reperfusion, vascular patency and graft perfusion were assessed using intraoperative Doppler ultrasonography. Primary abdominal closure was performed whenever this could be achieved without relevant tension or hemodynamic compromise. Otherwise, temporary abdominal closure with planned staged reconstruction was used. Postoperative care was provided according to standardized institutional protocols by a multidisciplinary team of pediatric transplant surgeons, hepatologists, anesthesiologists, and intensive care specialists.

### 2.6 Statistical Analysis

Categorical variables are presented as numbers and percentages and were compared using the chi‐square test or Fisher's exact test, as appropriate. Continuous variables were assessed for distribution and are presented as mean with standard deviation for approximately normally distributed data or median with interquartile range for non‐normally distributed data. Comparisons between LLS and WLG recipients were performed using the independent‐samples *t*‐test or Mann–Whitney *U* test, as appropriate.

Logistic regression analyses were performed to evaluate factors associated with bile leakage, biliary anastomotic stricture, major postoperative complications, and unplanned surgical reintervention. Variables considered clinically relevant and variables with a *p* value < 0.10 in univariable analysis were considered for inclusion in the multivariable models.

Graft and patient survival were estimated using the Kaplan–Meier method and compared between graft groups using the log‐rank test. Recipients were censored at the date of last documented follow‐up. All tests were two‐sided, and a *p* value < 0.05 was considered statistically significant. No adjustment for multiple comparisons was performed.

Statistical analyses were performed using IBM SPSS Statistics, version 22.0 (IBM Corp., Armonk, NY, USA), and GraphPad Prism (GraphPad Software, Boston, MA, USA).

## 3 Results

### 3.1 Recipient and Donor Characteristics

Of the 120 pediatric LT procedures performed during the study period, 95 met the inclusion criteria. The final cohort comprised 56 LLS recipients and 39 WLG recipients (Figure 1).

**FIGURE 1:**
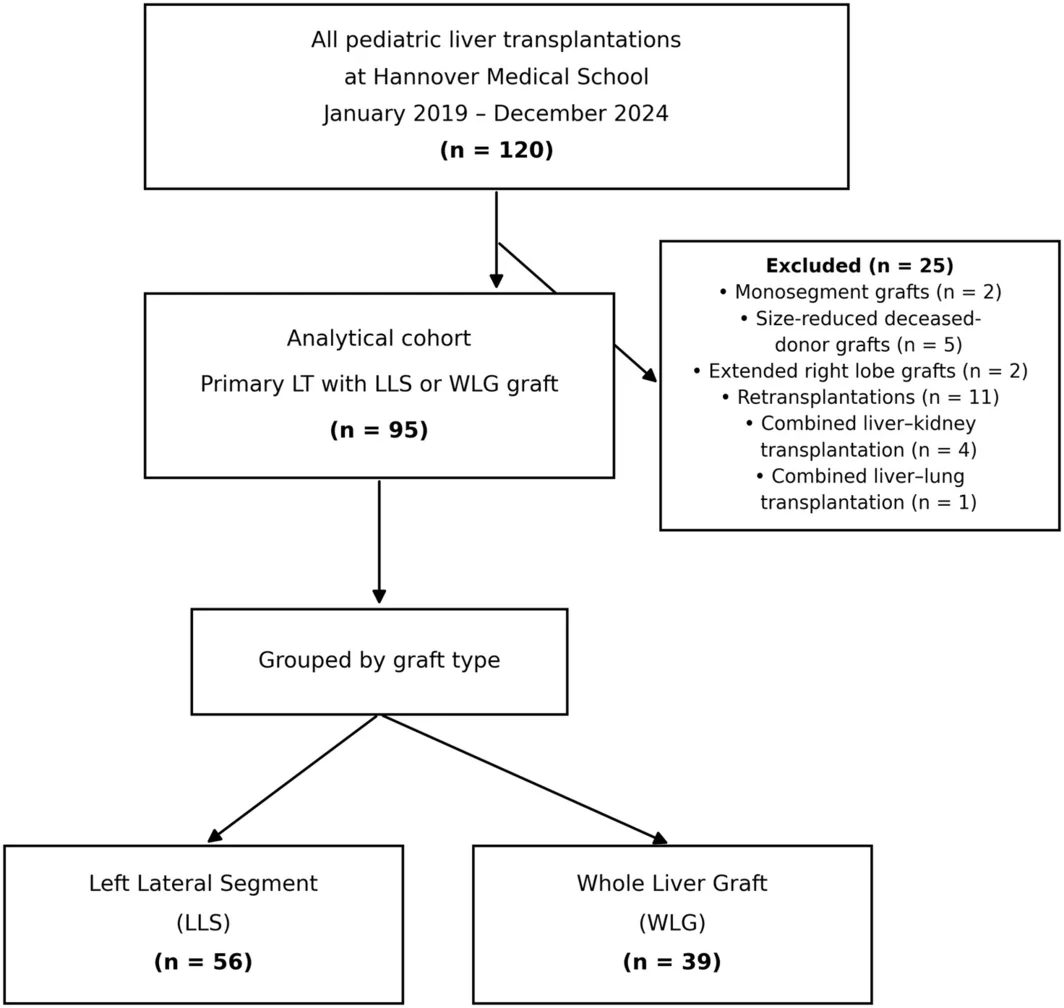
Study flowchart illustrating patient selection. LLS: left lateral segment graft; WLG: whole liver graft.

LLS recipients were significantly younger and smaller than WLG recipients. Median age at LT was 14.5 months in the LLS group and 70 months in the WLG group, while median body weight was 8 and 21 kg, respectively (both *p* < 0.001). Accordingly, 34 LLS recipients (61%) weighed < 10 kg compared with five WLG recipients (13%; *p* < 0.001). Recipient sex, underlying diagnosis, pretransplant clinical conditions, and high‐urgency status did not differ significantly between groups (Table 1).

**TABLE 1:** Recipient and donor demographic data (IQR: interquartile range; SD: standard deviation).

	Total	Recipients with LLS grafts	Recipients with whole organ grafts	*p*
Recipient characteristics				
Recipient, *n* (%)	95 (100)	56 (59)	39 (41)	
Sex, *n* (%)				
Male	49 (52)	32 (57)	17 (44)	0.193
Female	46 (48)	24 (43)	22 (56)	
Age at transplantation (months), median (IQR)	24.5 (62)	14.5 (19.8)	70 (90)	< 0.001
Body weight (kg), median (IQR)	12 (12.8)	8 (7)	21 (21)	< 0.001
Height (m), median (IQR)	0.9 (0.4)	0.7 (0.3)	1.1 (0.5)	< 0.001
Body weight < 10 kg, *n* (%)	39 (41)	34 (61)	5 (13)	< 0.001
Pre‐transplantation blood results				
PELD score, median (IQR)	28 (12)	28 (8)	30 (12)	0.138
MELD score (> 12 years), median (IQR)	16.5 (15.3)		16.5 (15.3)	
Sodium (mmol/L), mean (SD)	138 (4)	138 (5)	139 (3)	0.252
INR, mean (SD)	1.4 (0.7)	1.4 (0.7)	1.3 (0.6)	0.415
Total bilirubin (μmol/L), mean (SD)	177 (292)	230 (350)	51 (230)	0.014
Creatinine (μmol/L), mean (SD)	22 (13)	20 (9)	28 (18)	< 0.001
Platelet count (G/L), mean (SD)	118 (108)	116 (135)	118 (118)	0.176
Serum albumin (g/L), mean (SD)	32 (9)	30 (9)	34 (10)	0.257
Pre‐transplantation conditions, *n* (%)				
Portal vein thrombosis	5 (5)	5 (9)	0	0.055
Congenital heart disease	12 (13)	8 (14)	4 (10)	0.561
Hepatopulmonary syndrome	3 (3)	2 (4)	1 (3)	0.782
Kasai procedure	40 (42)	26 (46)	14 (36)	0.306
Pre‐transplantation dialysis	2 (2)	2 (4)	0	0.233
Recipient diagnosis, *n* (%)				
Biliary Atresia (BA)	40 (42)	26 (46)	14 (36)	0.191
Other cholestatic diseases	20 (21)	8 (14)	12 (31)	
Metabolic disorders	12 (13)	7 (13)	5 (13)	
Acute liver failure	10 (11)	5 (9)	5 (13)	
Tumors	8 (8)	5 (9)	3 (8)	
Other indications	5 (5)	5 (9)	0	
High urgency status, *n* (%)	28 (30)	17 (30)	11 (28)	0.821
Donor characteristics				
Sex, *n* (%)				
Male	56 (59)	36 (64)	20 (51)	0.205
Female	39 (41)	20 (36)	19 (49)	
Donor age (years), median (IQR)	26 (25.8)	30 (12.3)	7 (23)	< 0.001
BMI kg/m^2^, median (IQR)	21.7 (4.8)	23.6 (4)	19 (4.7)	0.079
Donor type, *n* (%)				
Living donor	23 (24)	23 (41)	0	< 0.001
Deceased donor	72 (76)	33 (59)	39 (100)	
ABO‐incompatible transplantation, *n* (%)	9 (10)	8 (14)	1 (3)	0.055
Graft characteristics				
Graft weight (g), median (IQR)	353 (294)	305.5 (97.6)	727.4 (845)	< 0.001
GRWR (%), median (IQR)	3.4 (1.8)	3.4 (2.6)	3.3 (1.5)	0.815

*Note:* Categorical data are shown as *n* (%) and tested with Chi‐square. Non‐normal continuous data are shown as median (IQR) and tested with Mann–Whitney *U*. Normal data are shown as mean (SD) and tested with independent *t*‐test. GRWR: Graft‐recipient‐weight‐ratio.

Pretransplant laboratory parameters were largely comparable between groups. Total bilirubin was higher in LLS recipients (*p* = 0.014), whereas creatinine was higher in WLG recipients (*p* < 0.001). No significant differences were observed in the Pediatric End‐stage Liver Disease (PELD) score, serum sodium, international normalized ratio, platelet count, or serum albumin.

Donors of LLS grafts were older than donors of WLGs (*p* < 0.001). Of the 56 LLS grafts, 23 (41%) were obtained from living donors and 33 (59%) from deceased donors, whereas all WLGs were obtained from deceased donors. Graft weight was lower in the LLS group (*p* < 0.001), while GRWR was comparable between groups (median 3.4% vs. 3.3%, *p* = 0.815).

Median waiting time was numerically shorter among LLS recipients than among WLG recipients (60 vs. 188 days), although the difference did not reach statistical significance (*p* = 0.064).

### 3.2 Operative Characteristics

Operative time, warm ischemia time, estimated blood loss, and red blood cell (RBC) transfusion requirements did not differ significantly between groups (Table 2). Cold ischemia time was shorter in the LLS group than in the WLG group (median 525.5 vs. 576 min, *p* = 0.031).

**TABLE 2:** Surgical and intraoperative parameters, including surgical and technical details.

	Total	Recipients with LLS grafts	Recipients with whole organ grafts	*p*
Surgical details				
Operative time (min), median (IQR)	255 (85.3)	256 (87.8)	255 (86)	0.865
Cold ischemia time (min), median (IQR)	561 (247.8)	525 (340)	576 (153)	0.031
Warm ischemia time (min), median (IQR)	51 (12.8)	50.5 (12.3)	53 (12)	0.224
Estimated blood loss (mL), median (IQR)	500 (500)	500 (487.5)	500 (750)	0.515
Red blood cell transfusion (units), median (IQR)	2 (2)	2 (1)	2 (2)	0.813
Technical details				
Number of bile duct anastomosis, *n* (%)				0.120
1	87 (92)	49 (86)	38 (97)	
2	7 (7)	6 (11)	1 (3)	
3	1 (1)	1 (2)	0	
Unknown	1 (1)	1 (2)	0	
Hepaticojejunostomy, *n* (%)	76 (80)	56 (100)	20 (51)	< 0.001
Duct‐to‐duct, *n* (%)	19 (20)	0	19 (49)	< 0.001
Use of interposition grafts, *n* (%)	19 (20)	16 (28)	3 (8)	0.230
Portal flow modulation, *n* (%)	2 (2)	2 (4)	0	0.510
Surgical management and revision procedures				
Primary abdominal closure, *n* (%)	22 (23)	4 (7)	18 (46)	< 0.001
Two‐step LT surgery, *n* (%)	69 (73)	48 (86)	21 (54)	< 0.001
Unplanned surgical reintervention, *n* (%)	27 (28)	24 (43)	3 (8)	< 0.001

*Note:* Categorical data are shown as *n* (%) and tested with Chi‐square. Non‐normal continuous data are shown as median (IQR) and tested with Mann–Whitney *U*. Normal data are shown as mean (SD) and tested with independent *t*‐test.

Biliary reconstruction differed substantially between groups. All LLS recipients underwent Roux‐en‐Y hepaticojejunostomy, whereas biliary reconstruction in WLG recipients consisted of either hepaticojejunostomy or duct‐to‐duct anastomosis. The number of biliary anastomoses, use of vascular interposition grafts, and portal flow modulation did not differ significantly between groups.

Primary abdominal closure was achieved in four LLS recipients (7%) and 18 WLG recipients (46%; *p* < 0.001). A planned two‐step LT procedure was performed in 48 LLS recipients (86%) and 21 WLG recipients (54%; *p* < 0.001).

### 3.3 Cumulative 90‐Day Morbidity and Unplanned Surgical Reinterventions

Cumulative postoperative morbidity within 90 days was higher after LLS transplantation. The median CCI was 53 in LLS recipients compared with 45 in WLG recipients (*p* = 0.002; Table 3).

**TABLE 3:** Postoperative complications and clinical outcomes in pediatric liver transplant recipients stratified by graft type (left lateral segment vs. whole liver graft).

	Total	Recipients with LLS grafts	Recipients with whole organ grafts	*p*
Postoperative complications				
CCI score, median (IQR)	50 (23)	53 (27)	45 (20)	0.002
Hepatic artery thrombosis, *n* (%)	5 (5)	3 (5)	2 (5)	1.000
Portal vein thrombosis, *n* (%)	7 (7)	6 (11)	1 (3)	0.234
Biliary complications, *n* (%)	30 (32)	17 (30)	13 (33)	0.759
Bile leak	11 (12)	10 (18)	1 (3)	0.024
Anastomotic biliary stricture, *n* (%)	16 (17)	5 (9)	11 (28)	0.014
Non‐anastomotic biliary stricture, *n* (%)	3 (3)	2 (4)	1 (3)	1.000
Bowel perforation, *n* (%)	3 (3)	3 (5)	0	0.266
Acute cellular rejection, *n* (%)	28 (30)	17 (30)	11 (28)	0.821
Postoperative dialysis, *n* (%)	5 (5)	4 (7)	1 (3)	0.645
Intra‐abdominal bleeding, *n* (%)	8 (9)	8 (15)	0	0.019
Retransplantation, *n* (%)	3 (3)	2 (4)	1 (3)	1.000
Postoperative outcomes and survival				
Graft loss, *n* (%)	6 (6)	4 (7)	2 (5)	1.000
Patient mortality, *n* (%)	6 (6)	4 (7)	2 (5)	1.000
Length of ICU stay (days), median (IQR)	10 (15)	11 (15.25)	5 (4‐24)	0.077
Length of hospital stay (days), median (IQR)	36 (23.75)	43 (32.25)	27	< 0.001
Patient survival at 1, 3, and 5 years, %		95%/92%/92%	97%/95%/95%	
Graft survival at 1, 3, and 5 years, %		96%/94%/89%	97%/94%/94%	
Follow‐up time (days), mean (SD)	1054 (544.4)	1010 (571.35)	1097 (519.3)	0.560
Waiting time (days), median (IQR)	70 (367.5)	60 (177.3)	188 (570)	0.064

*Note:* Values are presented as *n* (%) or median (interquartile range, IQR), unless otherwise specified. Statistical comparisons were performed between groups, with *p*‐values < 0.05 considered statistically significant.

Unplanned surgical reinterventions were required in 24 LLS recipients (43%) and three WLG recipients (8%; *p* < 0.001). Intra‐abdominal bleeding occurred in eight LLS recipients (15%) but in no WLG recipient (*p* = 0.019). Rates of acute cellular rejection and postoperative dialysis did not differ significantly between groups.

### 3.4 Biliary Complications

The overall incidence of biliary complications did not differ significantly between LLS and WLG recipients (30% vs. 33%, *p* = 0.759). However, the pattern and timing of biliary complications differed between groups. Within 90 days after LT, bile leakage occurred in ten LLS recipients (18%) and one WLG recipient (3%; *p* = 0.024). During the entire follow‐up period, anastomotic biliary strictures were diagnosed in five LLS recipients (9%) and 11 WLG recipients (28%; *p* = 0.014). Non‐anastomotic biliary strictures were uncommon and occurred at similar rates in both groups.

Among WLG recipients with a body weight comparable to the LLS cohort (*n* = 11, < 15 kg), 90‐day morbidity was similar to or higher than in the WLG cohort overall (median CCI 68.8 vs. 44.7; hepatic artery thrombosis 18% vs. 5%) (Supplementary Table 1).

Management strategies reflected these different complication patterns (Table 4). Bile leaks after LLS transplantation were predominantly managed by unplanned surgical reintervention or radiological drainage. Anastomotic strictures after WLG transplantation were most commonly treated by endoscopic retrograde cholangiopancreatography (ERCP).

**TABLE 4:** Management of postoperative complications.

Complication	Initial management	Total	Recipients with LLS grafts	Recipients with whole organ grafts
HAT	Surgical reintervention	4	2	2
	Systemic thrombolysis	1	1	0
PVT	Radiological intervention	4	3	1
	Surgical revision	3	3	0
Bile leak	ERCP	1	0	1
	Radiological drainage	3	3	0
	Surgical reintervention	5	5	0
	Observation	1	1	0
Anastomotic stricture	ERCP	10	0	10
	Radiological drainage	1	1	0
	Surgical reintervention	4	3	1
	Observation	1	1	0
Non‐anastomotic stricture	ERCP	1	0	1
	Surgical reintervention	2	2	0

Abbreviations: ERCP: endoscopic retrograde cholangiopancreatography; HAT: hepatic artery thrombosis; LLS: left lateral segment graft; PVT: portal vein thrombosis; WLG: whole liver graft.

### 3.5 Exploratory Regression Analyses

#### 3.5.1 Bile Leakage

In univariable logistic regression analysis, the number of intraoperative RBC units transfused was associated with bile leakage, with an odds ratio (OR) of 1.37 per transfused unit (*p* = 0.035). LLS graft type and increasing donor age were associated with higher estimated odds of bile leakage, although neither association reached statistical significance.

In multivariable analysis, RBC transfusion remained associated with bile leakage (OR 1.50, 95% confidence interval [CI] 1.05–2.14, *p* = 0.026; Table 5). The estimated association with LLS graft type was imprecise and did not reach statistical significance (OR 7.79, 95% CI 0.75–80.59, *p* = 0.085).

**TABLE 5:** Exploratory multivariable logistic regression analyses of factors associated with postoperative outcomes.

Variable	*p*‐value	OR	95% CI
Bile leak			
Graft type (LLS vs. WLG)	0.085	7.79	0.75–80.59
Donor age (years)	0.257	1.04	0.97–1.13
RBC transfusion (units)	0.026	1.50	1.05–2.14
Biliary anastomotic stricture			
Graft type (LLS vs. WLG)	0.071	0.29	0.08–1.09
Recipient age (months)	0.018	1.014	1.002–1.027
Graft weight (g)	0.041	1.002	1.000–1.003
Hepaticojejunostomy	0.002	0.18	0.06–0.58
Revisions			
Graft type (LLS vs. WLG)	0.012	6.21	1.49–25.88
Recipient weight (kg)	0.314	0.96	0.89–1.04
GRWR (%)	0.041	1.39	1.01–1.92
Hepaticojejunostomy	0.048	7.14	1.02–49.87

Abbreviations: CI: confidence interval; Exp(B): exponentiated regression coefficient (odds ratio); GRWR: graft‐to‐recipient weight ratio; LLS: left lateral segment graft; RBC: red blood cell; WLG: whole liver graft.

#### 3.5.2 Anastomotic Biliary Stricture

In univariable analysis, LLS graft type was associated with lower odds of anastomotic biliary stricture than WLG transplantation (OR 0.25, *p* = 0.018). Increasing recipient age, recipient weight, and graft weight were associated with higher odds of stricture formation, whereas hepaticojejunostomy was associated with lower odds.

In multivariable analysis, increasing recipient age (OR 1.014 per month, 95% CI 1.002–1.027, *p* = 0.018) and graft weight (OR 1.002 per gram, 95% CI 1.000–1.003, *p* = 0.041) remained associated with anastomotic biliary stricture. Hepaticojejunostomy was associated with lower odds of stricture formation (OR 0.18, 95% CI 0.06–0.58, *p* = 0.002; Table 5).

#### 3.5.3 Major Postoperative Complications

In multivariable analysis, a higher PELD score was associated with major postoperative complications (OR 1.11, 95% CI 1.01–1.23, *p* = 0.035). Hepaticojejunostomy was associated with lower odds of major postoperative complications than duct‐to‐duct reconstruction (OR 0.02, 95% CI 0.00–0.85, *p* = 0.041). Graft type was not associated with major postoperative complications (OR 1.48, 95% CI 0.31–7.19, *p* = 0.624), while lower recipient weight showed a nonsignificant association (OR 0.86, 95% CI 0.73–1.01, *p* = 0.065; Table 5).

#### 3.5.4 Unplanned Surgical Reinterventions and GRWR

In univariable analysis, LLS graft type, younger recipient age, lower recipient weight, lower graft weight, increasing GRWR, and hepaticojejunostomy were associated with unplanned surgical reintervention. In multivariable analysis, LLS graft type (OR 6.21, 95% CI 1.49–25.88, *p* = 0.012), increasing GRWR (OR 1.39 per percentage point, 95% CI 1.01–1.92, *p* = 0.041), and hepaticojejunostomy (OR 7.14, 95% CI 1.02–49.87, *p* = 0.048) remained associated with unplanned surgical reintervention (Table 5).

In a separate univariable analysis, increasing GRWR was associated with unplanned surgical reintervention (OR 1.32, 95% CI 1.00–1.74, *p* = 0.047), but not with major postoperative complications (OR 0.99, 95% CI 0.73–1.37, *p* = 0.982) or primary abdominal closure (OR 0.77, 95% CI 0.54–1.09, *p* = 0.145). When GRWR was categorized as < 3%, 3%–5%, and > 5%, the proportion of unplanned surgical reinterventions was highest among LLS recipients with a GRWR > 5%, although the difference was not statistically significant.

#### 3.5.5 Vascular Complications

HAT occurred in three LLS recipients (5%) and two WLG recipients (5%; *p* = 1.000). Four recipients underwent unplanned surgical reintervention, while one LLS recipient was successfully treated with systemic thrombolysis.

Two of the three LLS recipients with HAT also developed PVT, resulting in graft loss and retransplantation. One of these recipients subsequently died.

PVT occurred in six LLS recipients (11%) and one WLG recipient (3%; *p* = 0.234). Among the four LLS recipients with isolated PVT, three underwent radiological recanalization combined with anticoagulation, and one underwent surgical embolectomy. The WLG recipient with PVT underwent radiological recanalization (Tables 3 and 4).

In univariable analysis, prior Kasai procedure was associated with higher odds of postoperative PVT (OR 9.53, 95% CI 1.10–82.64, *p* = 0.041). Lower recipient weight, shorter cold ischemia time, and longer warm ischemia time showed nonsignificant associations with PVT. The small number of HAT events precluded a robust multivariable analysis.

### 3.6 Postoperative Recovery and Survival

Graft loss occurred in four LLS recipients (7%) and two WLG recipients (5%; *p* = 1.000). Patient mortality was 7% in the LLS group and 5% in the WLG group (*p* = 1.000).

Median hospital stay was longer among LLS recipients than among WLG recipients (43 vs. 27 days, *p* < 0.001). Median ICU stay was 11 days after LLS transplantation and 5 days after WLG transplantation, although the difference did not reach statistical significance (*p* = 0.077).

No statistically significant difference in graft survival was detected between groups (Figure 2A; log‐rank *p* = 0.645). Estimated graft survival at 1, 3, and 5 years was 96%, 94%, and 89% after LLS transplantation and 97%, 94%, and 94% after WLG transplantation.

**FIGURE 2:**
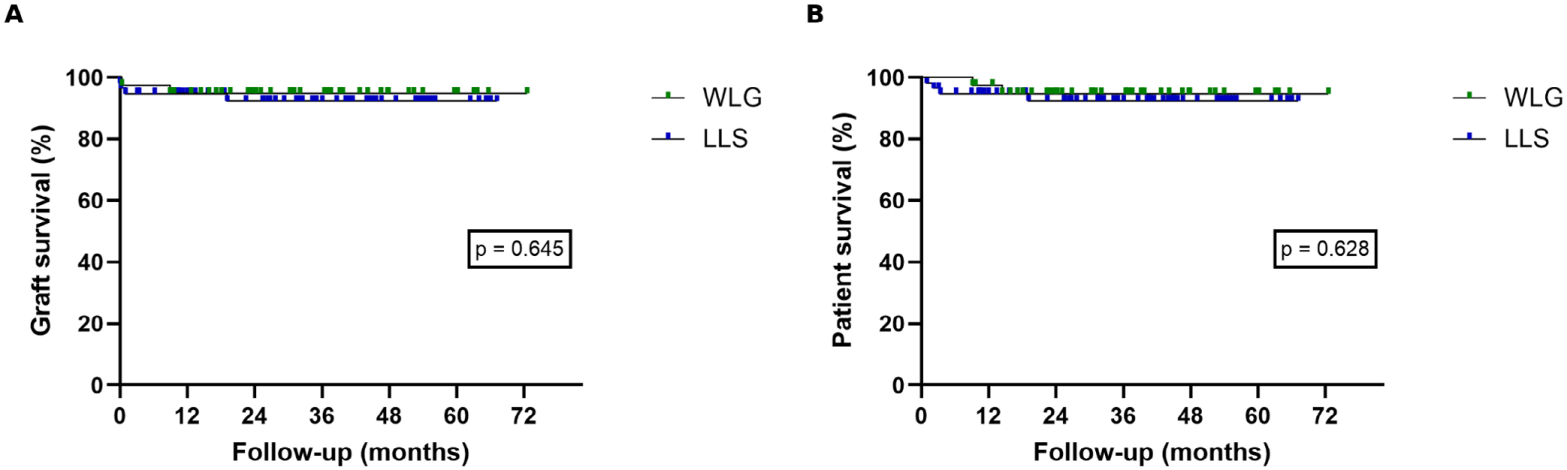
Kaplan–Meier curves for graft survival (A) and patient survival (B) in recipients of whole liver grafts (WLG) and left lateral segment grafts (LLS). Neither graft survival nor patient survival differed significantly between groups (graft survival: Log‐rank *p* = 0.645; patient survival: Log‐rank *p* = 0.628).

Patient survival also did not differ significantly between groups (Figure 2B; log‐rank *p* = 0.628). Estimated patient survival at 1, 3, and 5 years was 95%, 92%, and 92% in the LLS group and 97%, 95%, and 95% in the WLG group. Follow‐up duration was comparable between groups.

## 4 Discussion

In our cohort, LLS grafts were used predominantly in infants and small children, whereas WLGs were transplanted mainly into older and larger recipients, consistent with established pediatric transplant practice. These graft types should therefore be regarded as complementary strategies for distinct clinical populations rather than competing techniques for interchangeable candidates. In this retrospective single‐center cohort, LLS transplantation was associated with greater cumulative morbidity within 90 days than WLG transplantation, as reflected by higher CCI scores. This increased postoperative burden was characterized by higher rates of bile leakage, intra‐abdominal bleeding, and unplanned surgical reintervention. Although the overall incidence of biliary complications was similar between groups, bile leakage was more frequent after LLS transplantation, whereas anastomotic biliary strictures occurred more frequently after WLG transplantation. The greater early morbidity after LLS transplantation was accompanied by a longer hospital stay but not by a detected difference in graft loss, retransplantation, or graft and patient survival. Taken together, these findings indicate a greater cumulative early postoperative burden after LLS transplantation without a detectable disadvantage in graft or patient survival during the available follow‐up. By incorporating all complications occurring within 90 days, the CCI captures the cumulative postoperative burden of repeated events and interventions that may not be reflected by reporting individual complication rates alone [12]. In our cohort, higher CCI scores among LLS recipients were accompanied by more frequent unplanned surgical reinterventions and longer hospital stays, underscoring the clinical relevance of the observed difference.

To our knowledge, this is the first study to apply the CCI to compare cumulative early postoperative morbidity between LLS and WLG recipients in a pediatric cohort. Previous reports compared individual isolated complications [8, 9, 10, 11], whereas the CCI captures their cumulative, severity‐weighted burden. A similar CCI‐based comparison in adults found no significant difference between whole and split liver grafts [8].

In the exploratory regression analysis, increasing GRWR was associated with higher odds of unplanned surgical reintervention. This association may reflect the technical challenges of graft–recipient size mismatch, including increased abdominal tension in small recipients. This interpretation is consistent with the findings of Goldaracena et al., who reported that a high GRWR in pediatric LLS transplantation was associated with delayed abdominal closure but not with increased complication rates or impaired survival [2]. Zakaria et al. reported that more than half of large‐for‐size LLS recipients required temporary abdominal closure, while vascular complications, biliary complications, and survival remained comparable to standard‐sized grafts [9]. In our cohort, however, categorization of GRWR into clinically relevant ranges did not identify a significant threshold associated with unplanned surgical reintervention.

The observed associations between graft type and postoperative outcomes cannot be interpreted independently of the clinical and procedural differences between the two groups. All WLGs were obtained from deceased donors, whereas 41% of LLS grafts originated from living donors. Differences in donor selection, graft procurement, and ischemia times may therefore have contributed to the observed differences in postoperative outcomes. Recipient age and size also differed markedly between groups and may themselves influence surgical complexity and the postoperative course. Accordingly, the present study compares two clinically distinct graft strategies rather than isolating the effect of graft type alone [2, 9, 14, 15].

This may also explain findings in the size‐matched WLG subgroup (Supplementary Table 1), where recipients weighing < 15 kg showed a morbidity pattern closer to that of the LLS cohort despite receiving a whole graft. Recipient age, size, and underlying disease, rather than graft type alone, contributes to early postoperative morbidity.

The higher incidence of bile leakage after LLS transplantation is consistent with previous reports on technical variant grafts [10, 11]. Potential explanations include bile leakage from the transection surface, the small caliber of the bile ducts, and the technical complexity of biliary reconstruction in small pediatric recipients [16]. Conversely, the higher incidence of anastomotic biliary strictures after WLG transplantation has been less consistently described in the existing literature. In our cohort, this finding may partly reflect differences in biliary reconstruction, as duct‐to‐duct anastomosis was confined to WLG recipients and hepaticojejunostomy was associated with lower odds of anastomotic stricture. At the same time, hepaticojejunostomy was associated with higher odds of unplanned surgical reintervention, likely reflecting differences in recipient characteristics and operative complexity rather than a direct adverse effect of the reconstruction technique. The association between intraoperative RBC transfusion and bile leakage should likewise be interpreted cautiously, as transfusion may primarily represent a marker of operative complexity and blood loss rather than a direct cause of biliary complications. These associations should be interpreted with caution because graft type, recipient size, and biliary reconstruction technique were closely interrelated and the number of individual events was limited.

The increased early morbidity associated with LLS transplantation should be considered in the context of its established role in expanding graft availability for infants and small children [3, 4, 5]. Contemporary registry studies indicate that the historical survival disadvantage associated with technical variant grafts has diminished substantially in modern pediatric transplant practice [11, 19]. In our cohort, waiting time was numerically shorter among LLS recipients, although the difference did not reach statistical significance. Because the analysis included only children who ultimately underwent LT, it does not allow conclusions regarding overall access to transplantation or waitlist mortality. The observed waiting‐time difference should therefore be regarded as descriptive and not as evidence that LLS transplantation improved access to LT in this cohort. These findings are consistent with recent United States registry data showing that greater center‐level use of technical variant grafts, including LLS grafts, is associated with improved waitlist outcomes without evidence of worse recipient outcomes [17].

Overall, we consider LLS grafts to remain an important and necessary option when no suitably size‐matched whole graft is available in time. However, families should be informed that this comes with greater early morbidity, including a higher rate of unplanned reoperations and a longer hospital stay, without an increased risk of graft loss or death. This trade‐off should be discussed openly with families and considered in perioperative planning, rather than seen as a reason to avoid LLS grafts.

This study has several limitations, primarily its retrospective single‐center design, which introduces the potential for selection bias and limits the generalizability of the findings. The LLS and WLG groups differed substantially in recipient age and size, donor type, and biliary reconstruction technique, limiting the extent to which observed outcome differences can be attributed to graft type alone. The limited number of biliary and vascular complications, graft losses, and deaths restricted the robustness of the multivariable analyses and resulted in wide confidence intervals for several estimates. The study was not designed or powered to demonstrate equivalence in graft or patient survival between the two groups. Finally, the duration of follow‐up was insufficient to assess outcomes extending into adolescence and adulthood. All procedures were performed by the same experienced team throughout the study period. A chronological comparison found no significant reduction in LLS‐related morbidity over time, arguing against a simple learning‐curve explanation for the observed differences (Supplementary Table 2).

## 5 Conclusion

In this cohort, use of the CCI as a novel, cumulative morbidity score identified significantly greater 90‐day postoperative burden after LLS transplantation, characterized by higher rates of bile leakage, intra‐abdominal bleeding, unplanned surgical reintervention, and prolonged hospitalization. Despite this greater early postoperative burden, no statistically significant differences in graft loss, retransplantation, or graft and patient survival were detected during the available follow‐up.

## Author Contributions

H.L., C.J.V.B., and E.‐D.P. conceived the study. H.L. collected and analyzed the data and drafted the manuscript. C.J.V.B. and M.S. supervised the study and revised the manuscript. V.M., S.A., J.F., T.‐Y.T‐and T.L. contributed to data collection and manuscript editing. N.R., M.Q., and P.F. contributed to surgical care and manuscript revision. E.‐D.P. and U.B. contributed to pediatric hepatology expertise, patient care, and manuscript revision. All authors reviewed and approved the final manuscript.

## Funding

This work was supported by PRACTIS Clinician Scientist Program, ME 3696/3.

## Disclosure

M.S. reports consulting and/or research support from Merck Serono GmbH, Bayer AG, ERBE Elektromedizin GmbH, Amgen Inc., AstraZeneca, Takeda Pharmaceutical Limited, Bowa Medical, Corza Medical, Medtronic GmbH, Johnson & Johnson Medical GmbH, Intuitive Surgical Inc., Chiesi GmbH, OrganOx, and Baxter International Inc. C.J.V.B. is part of the scientific advisory board for Bridge‐to‐Life Ltd.

## Conflicts of Interest

The authors declare no conflicts of interest.

## Supporting information


**Supplementary Table 1.** Descriptive 90‐day morbidity in the full LLS and WLG cohorts and in the subgroups of each restricted to recipients weighing < 15 kg (CCI: Comprehensive Complication Index; HAT: hepatic artery thrombosis; IQR: interquartile range; PVT: portal vein thrombosis). The WLG < 15 kg subgroup was defined to cover the interquartile range of LLS recipient body weight with a modest margin. Given the small subgroup size, values are presented descriptively and were not subjected to formal statistical comparison.
**Supplementary Table 2**. Chronological comparison of 90‐day morbidity, 2019–2021 versus 2022–2024, within the LLS and WLG groups (CCI: Comprehensive Complication Index; IQR: interquartile range). *p*‐values: Mann–Whitney *U* test for CCI; chi‐square test when all expected cell counts were 5, otherwise Fisher's exact test. All procedures throughout the study period were performed by the same experienced pediatric transplant team.

## Data Availability

The data that support the findings of this study are available on request from the corresponding author. The data are not publicly available due to privacy or ethical restrictions.
