# Supplementary material for: Early Morbidity and Survival After Left Lateral Segment Versus Whole Liver Graft Transplantation in Children
Source: Pediatr Transplant. 2026 Oct 20;30(10):e70501. doi: 10.1111/petr.70501 (PMC13681243; doi:10.1111/petr.70501)
Supplement: Supplementary file 1 [file PETR-30-e70501-s001.docx]

*Supplementary Table 1. Descriptive 90-day morbidity in the full LLS and WLG cohorts and in the subgroups of each restricted to recipients weighing <15 kg (IQR: Interquartile Range; CCI: Comprehensive Complication Index; HAT: Hepatic artery thrombosis; PVT: Portal vein thrombosis. The WLG <15 kg subgroup was defined to cover the interquartile range of LLS recipient body weight with a modest margin. Given the small subgroup size, values are presented descriptively and were not subjected to formal statistical comparison.*

|  | **LLS, full cohort** | **LLS, <15 kg** | **WLG, <15 kg** | **WLG, full cohort** |
| --- | --- | --- | --- | --- |
| n | 56 | 44 | 11 | 39 |
| Age, months, median (IQR) | 13.5 (7.0–24.3) | 8.0 (7.0–18.5) | 20.0 (7.5–32.5) | 70.0 (34.5–123.5) |
| Weight, kg, median (IQR) | 8.0 (6.2–12.3) | 7.0 (6.0–9.5) | 11.8 (7.0–12.3) | 20.8 (13.8–32.2) |
| CCI, median (IQR) | 53.2 (47.9–71.5) | 53.2 (48.2–67.3) | 68.8 (40.7–79.3) | 44.7 (39.5–57.9) |
| Biliary complication, any, n (%) | 17 (30) | 12 (27) | 3 (27) | 13 (33) |
| Bile leakage, n (%) | 10 (18) | 6 (14) | 0 (0) | 1 (3) |
| Anastomotic stricture, n (%) | 5 (9) | 4 (9) | 3 (27) | 11 (28) |
| Hepatic artery thrombosis, n (%) | 3 (5) | 3 (7) | 2 (18) | 2 (5) |
| Portal vein thrombosis, n (%) | 6 (11) | 5 (11) | 1 (9) | 1 (3) |
| Bleeding, n (%) | 8 (14) | 6 (14) | 0 (0) | 0 (0) |
| Graft loss, n (%) | 4 (7) | 4 (9) | 0 (0) | 2 (5) |
| Mortality, n (%) | 4 (7) | 3 (7) | 0 (0) | 2 (5) |

*Supplementary Table 2. Chronological comparison of 90-day morbidity, 2019–2021 versus 2022–2024, within the LLS and WLG groups (IQR: Interquartile Range; CCI: Comprehensive Complication Index. P-values: Mann-Whitney U test for CCI; chi-square test when all expected cell counts were ≥5, otherwise Fisher's exact test. All procedures throughout the study period were performed by the same experienced pediatric transplant team.*

|  | **LLS, 2019–2021** | **LLS, 2022–2024** | **LLS, p-value** | **WLG, 2019–2021** | **WLG, 2022–2024** | **WLG, p-value** |
| --- | --- | --- | --- | --- | --- | --- |
| n | 36 | 20 | – | 24 | 15 | – |
| CCI, median (IQR) | 52.8 (46.5–67.3) | 57.2 (48.3–78.0) | 0.521 | 44.8 (38.8–64.2) | 44.7 (39.5–47.0) | 0.470 |
| Biliary complication, any, n (%) | 9 (25) | 8 (40) | 0.242 | 11 (46) | 2 (13) | 0.036 |
| Bile leakage, n (%) | 4 (11) | 6 (30) | 0.142 | 1 (4) | 0 (0) | – |
| Anastomotic stricture, n (%) | 3 (8) | 2 (10) | – | 9 (38) | 2 (13) | – |
| Hepatic artery thrombosis, n (%) | 3 (8) | 0 (0) | 0.545 | 1 (4) | 1 (7) | – |
| Portal vein thrombosis, n (%) | 4 (11) | 2 (10) | – | 1 (4) | 0 (0) | – |
| Bleeding, n (%) | 6 (17) | 2 (10) | 0.697 | 0 (0) | 0 (0) | – |
| Graft loss, n (%) | 4 (11) | 0 (0) | – | 1 (4) | 1 (7) | – |
| Mortality, n (%) | 3 (8) | 1 (5) | – | 2 (8) | 0 (0) | – |
